# Supplementary material for: Network analysis of mental well-being, psychological inflexibility, and psychopathological symptoms in individuals seeking online psychological support
Source: PLoS One. 2026 Sep 23;21(9):e0344411. doi: 10.1371/journal.pone.0344411 (PMC13600521; doi:10.1371/journal.pone.0344411)
Supplement: Supplementary file 1 [file pone.0344411.s003.docx]

**Reviewers and editor comments**

| **Editor comments** | **Response to editor or reviewer** |
| --- | --- |
| Please ensure that your manuscript meets PLOS ONE's style requirements, including those for file naming. The PLOS ONE style templates can be found at  [https://journals.plos.org/plosone/s/file?id=wjVg/PLOSOne_formatting_sample_main_body.pdf](https://nam04.safelinks.protection.outlook.com/?url=https%3A%2F%2Fjournals.plos.org%2Fplosone%2Fs%2Ffile%3Fid%3DwjVg%2FPLOSOne_formatting_sample_main_body.pdf&data=05%7C02%7Cleivy%40tec.mx%7Ca4f6b4aa0f504a14174c08dec4768860%7Cc65a3ea60f7c400b89345a6dc1705645%7C0%7C0%7C639164210928007095%7CUnknown%7CTWFpbGZsb3d8eyJFbXB0eU1hcGkiOnRydWUsIlYiOiIwLjAuMDAwMCIsIlAiOiJXaW4zMiIsIkFOIjoiTWFpbCIsIldUIjoyfQ%3D%3D%7C0%7C%7C%7C&sdata=KT3xODgW0CuiVFLMZKrtX%2F5oCmmEp1%2FgdVaulp2P%2B8A%3D&reserved=0)  and  [https://journals.plos.org/plosone/s/file?id=ba62/PLOSOne_formatting_sample_title_authors_affiliations.pdf](https://nam04.safelinks.protection.outlook.com/?url=https%3A%2F%2Ftrack.editorialmanager.com%2FCL0%2Fhttps%3A%252F%252Fjournals.plos.org%252Fplosone%252Fs%252Ffile%253Fid%3Dba62%252FPLOSOne_formatting_sample_title_authors_affiliations.pdf%2F1%2F010f019e95ba871a-a8d820c4-cd1d-4c1a-8208-57ea373bf0bc-000000%2F-wBS-3iZN0XRRBoFmJE842fzSyIPqzCA5MRz9d5gXPU%3D258&data=05%7C02%7Cleivy%40tec.mx%7Ca4f6b4aa0f504a14174c08dec4768860%7Cc65a3ea60f7c400b89345a6dc1705645%7C0%7C0%7C639164210928024422%7CUnknown%7CTWFpbGZsb3d8eyJFbXB0eU1hcGkiOnRydWUsIlYiOiIwLjAuMDAwMCIsIlAiOiJXaW4zMiIsIkFOIjoiTWFpbCIsIldUIjoyfQ%3D%3D%7C0%7C%7C%7C&sdata=hUpW30mPSlFOWM1VyDCdKA%2BC8EPsc%2BqBUovhCX61AIk%3D&reserved=0) | We have reviewed the manuscript and ensured that it complies with the PLOS ONE's style requirements. |
| We note that there is identifying data in the Supporting Information file “S1-Data.xlsx”. Due to the inclusion of these potentially identifying data, we have removed this file from your file inventory. Prior to sharing human research participant data, authors should consult with an ethics committee to ensure data are shared in accordance with participant consent and all applicable local laws.  Data sharing should never compromise participant privacy. It is therefore not appropriate to publicly share personally identifiable data on human research participants. The following are examples of data that should not be shared:  -Name, initials, physical address  -Ages more specific than whole numbers  -Internet protocol (IP) address  -Specific dates (birth dates, death dates, examination dates, etc.)  -Contact information such as phone number or email address  -Location data  -ID numbers that seem specific (long numbers, include initials, titled “Hospital ID”) rather than random (small numbers in numerical order)  Data that are not directly identifying may also be inappropriate to share, as in combination they can become identifying. For example, data collected from a small group of participants, vulnerable populations, or private groups should not be shared if they involve indirect identifiers (such as sex, ethnicity, location, etc.) that may risk the identification of study participants.  Additional guidance on preparing raw data for publication can be found in our Data Policy (<https://journals.plos.org/plosone/s/data-availability#loc-human-research-participant-data-and-other-sensitive-data>) and in the following article: <http://www.bmj.com/content/340/bmj.c181.long>.  Please remove or anonymize all personal information (<specific identifying information in file to be removed>), ensure that the data shared are in accordance with participant consent, and re-upload a fully anonymized data set. Please note that spreadsheet columns with personal information must be removed and not hidden as all hidden columns will appear in the published file. | Thanks for this observation. We noticed that the specific ages were included and have been replaced with age ranges. For example, 18 to 30, 31 to 40, and so on.  We also noticed that 5 participants were under 18, and they were removed from the database.  None of the other possible sensitive information was included in the previous database, such as names, long IDs, IPs, etc. The updated file is included in this submission. |
| Your ethics statement should only appear in the Methods section of your manuscript. If your ethics statement is written in any section besides the Methods, please move it to the Methods section and delete it from any other section. Please ensure that your ethics statement is included in your manuscript, as the ethics statement entered into the online submission form will not be published alongside your manuscript. | The ethics statement only appears in the Methods section |
| Please include captions for your Supporting Information files at the end of your manuscript, and update any in-text citations to match accordingly. Please see our Supporting Information guidelines for more information: [http://journals.plos.org/plosone/s/supporting-information](http://track.editorialmanager.com/CL0/http:%2F%2Fjournals.plos.org%2Fplosone%2Fs%2Fsupporting-information/1/010f019e95ba871a-a8d820c4-cd1d-4c1a-8208-57ea373bf0bc-000000/3Oh7oupwEl8pW_UFd33uIzlVUxpAAV7M1kxEQaxM90A=258). | We have included the captions at the end of the manuscript. |
| If the reviewer comments include a recommendation to cite specific previously published works, please review and evaluate these publications to determine whether they are relevant and should be cited. There is no requirement to cite these works unless the editor has indicated otherwise. | We have carefully reviewed the reviewer’s comments and confirmed that they do not include any recommendations to cite specific previously published works. |
| **Reviewer 1** |  |
| Line 194: The statistical analysis section should be clearly divided into the following components: network estimation (Graphical LASSO), network visualization, centrality analysis, and network stability and accuracy (bootstrap) | Thank you for this helpful suggestion. We revised the statistical analysis section and reorganized it into clearly labelled subsections: data preparation and residualization, network estimation, network visualization, centrality analysis, network stability and accuracy, and gender-based network comparisons. |
| Line 196: The network structure was estimated using a Graphical Gaussian Model with L1 regularization via graphical LASSO. | We revised the wording accordingly. The section now states that the network structure was estimated using a Graphical Gaussian Model with L1 regularization via the graphical LASSO, as implemented in the bootnet package. |
| Line 199: Usually ≥10 participants per node for more stable estimates. | Thank you for noting this. We corrected the node count and clarified the participant-to-node ratio. The final network included 73 nodes and 941 participants, yielding a ratio of approximately 12.9 participants per node, which exceeds the commonly cited recommendation of at least 10 participants per node. |
| Line 204: The sentence could be improved e.g. The network was estimated using residualized item scores obtained from linear regression models such as age, gender, education, occupation, and marital status. | We revised this sentence for clarity. The section now states that item scores were residualized using linear regression models that included age, gender, education, occupation, and marital status as covariates, and that the resulting residualized item scores were used as input for the network analyses. |
| Table 1: The nodes represent which outcome measure is to be highlighted in the table footnote. | Thank you for this suggestion. We revised the Table 1 footnote to clarify that nodes represent individual item-level indicators from the outcome measures included in the network |
| The statistical test applied in Table 2 should be clearly stated in both the statistical analyses section and the corresponding table footnote. | We agree. We revised the Statistical Analysis section to specify the statistical tests used for Table 2 and added this information to the table footnote. The footnote now indicates which tests were used for categorical and continuous variables. |
| Table 2: The label ‘Overall’ is to be replaced with ‘Total’. Percentage values either based on row or column percentages sum to 100%, according to the intended focus of the table. | We revised Table 2 accordingly and replaced the label “Overall” with “Total.” |
| Other plots could be provided e.g. Edge-weight accuracy plot (bootstrap), bootstrapped difference test plot etc | We appreciate the recommendation and agree that there are additional visualizations available to demonstrate network stability. However, we have opted not to include the edge-weight accuracy plot in the main manuscript due to challenges with visualization readability, which is inherently tied to the number of connections. Furthermore, generating the bootstrapped difference plot was not feasible with our available computing resources; given that our network comprises 2,628 possible unique edges, the resulting object becomes excessively large because the plot compares edges against one another. Instead, we have included the CS-coefficients in the main manuscript to summarize the statistical information and provide evidence of the network’s stability. While we could have presented a figure with fewer comparisons, as this is an exploratory study, we chose not to prioritize specific comparisons arbitrarily. Consequently, we are unable to fulfil this suggestion, although we have addressed this point in the manuscript's results section (network stability). |
| Not all references conformed to the journal’s format.  <https://journals.plos.org/plosone/s/submission-guidelines#loc-references>  <https://www.nlm.nih.gov/bsd/uniform_requirements.html> | Thank you for your comment. We have carefully reviewed all references and revised them to ensure that they conform to the journal's required formatting. |
| **Reviewer 2** |  |
| #1. Study sample and rationale In the Discussion, the authors state: “The purpose of the present study was to identify key nodes related to stress, anxiety, depression, and psychological inflexibility that may play a central role in mental well-being in a sample of individuals seeking online psychological support. As far as we know, this research is the first to use network analysis to explore how these psychological constructs are connected within this group.” However, the characteristics of the study sample and the rationale for focusing on this population are not sufficiently explained in the Introduction. The authors should provide more detailed information about the sample, including the context in which participants sought online psychological support, and explain more clearly why examining these psychological constructs in this population is important. | We have expanded the introduction to provide a more detailed description of the study sample and the relevance of understanding their psychological experiences. The revised text highlights the growing use of digital mental health services and the need to better understand the factors associated with help-seeking in this context.  Furthermore, we have added a section highlighting the study's strengths and expanded the conclusion. |
| #2. Number of nodes In the Statistical Analyses section, the authors state that the analysis used 967 participants for a network of 74 nodes (N/p ≈ 13.1), exceeding recommended thresholds. However, in the legend for Figure 2, the authors state that the network contains 73 nodes, each representing an item, with edges indicating the magnitude and direction of partial correlations between pairs of nodes while controlling for all other analyzed variables. This discrepancy between 73 and 74 nodes may confuse readers. Please revise the relevant sections to ensure consistency and clearly state the correct number of nodes included in the network analysis. | Thank you for pointing this out. We have carefully reviewed the entire manuscript to ensure consistency and confirm that the network consists of 73 nodes throughout. |
| #3. Interpretation of negative Z-scores in Figure 3 In Figure 3, the authors state that standardized Z-scores, with a mean of 0 and a standard deviation of 1, indicate the importance of each item according to the calculated centrality metric, and that the further a point shifts to the right, the greater its importance. However, some items appear to have negative Z-scores and are located toward the left side of the figure. Please clarify how negative Z-scores should be interpreted in this context. | Thank you for this helpful comment. We revised the Figure 3 caption and accompanying text to clarify that Z-scores are standardized centrality values. Negative Z-scores indicate that a node has a centrality value below the network average for that metric; they do not indicate negative centrality or an inverse effect. We also clarified that points farther to the right indicate relatively higher centrality compared with other nodes. |
| #4. Network stability In the Network Stability section, the authors state that the correlation stability (CS) coefficients exceeded the recommended threshold of 0.5. Specifically, the coefficients were 0.75, 0.75, 0.67, and 0.44 for edges, strength, closeness, and betweenness, respectively. However, the CS coefficient for betweenness appears to be 0.44, which does not exceed the 0.5 threshold. Please clarify this point and provide a more detailed explanation of this issue. | Thank you for identifying this inconsistency. We corrected the text to indicate that the CS coefficients for edges, strength, and closeness exceeded the recommended 0.50 threshold, whereas the CS coefficient for betweenness was 0.44 and thus did not exceed it . We revised the interpretation accordingly and now state that betweenness was less stable and should be interpreted cautiously, with substantive conclusions based primarily on the more stable indices. |
| #5. References and citation support Some statements in the manuscript appear to require stronger citation support. Please review the manuscript carefully and add appropriate references where necessary. For example, the following statement in the Introduction should be supported by relevant literature: “It has been proposed that the concept of mental health or well-being refers to a stable state within a symptom network characterized by weak interconnections among its elements.” | Thank you for this suggestion. The following references have been added to support several statements throughout the manuscript, including the one in the introduction that the reviewer cited . In this case, we have cited the following reference:   - Philippe TJ, Sikder N, Jackson A, Koblanski ME, Liow E, Pilarinos A, et al. Digital Health Interventions for Delivery of Mental Health Care: Systematic and Comprehensive Meta-Review. JMIR Ment Health. 2022 May 1;9(5). doi:10.2196/35159 PubMed PMID: 35551058. - Lattie EG, Stiles-Shields C, Graham AK. An overview of and recommendations for more accessible digital mental health services. Nature Reviews Psychology 2022 1:2. 2022 Jan 26;1(2):87–100. doi:10.1038/s44159-021-00003-1 - Starvaggi I, Lorenzo-Luaces L. Psychotherapy Access Barriers and Interest in Digital Mental Health Interventions Among Adults With Treatment Needs: Survey Study. JMIR Ment Health. 2025;12. doi:10.2196/65356 PubMed PMID: 40168039. - Pretorius C, Chambers D, Coyle D. Young People’s Online Help-Seeking and Mental Health Difficulties: Systematic Narrative Review. J Med Internet Res. 2019 Nov 1;21(11). doi:10.2196/13873 PubMed PMID: 31742562. - Smith BM, Twohy AJ, Smith GS. Psychological inflexibility and intolerance of uncertainty moderate the relationship between social isolation and mental health outcomes during COVID-19. J Contextual Behav Sci. 2020 Oct 1;18:162. doi:10.1016/J.JCBS.2020.09.005 PubMed PMID: 32953435. - Borghouts J, Eikey E, Mark G, De Leon C, Schueller SM, Schneider M, et al. Barriers to and Facilitators of User Engagement With Digital Mental Health Interventions: Systematic Review. J Med Internet Res. 2021 Mar 1;23(3). doi:10.2196/24387 PubMed PMID: 33759801. - Van Den Noortgate M, Morrens M, Van Hemert AM, Schoevers RA, Penninx BWJH, Giltay EJ. Unveiling Transitions in Disease States: Study of Depressive and Anxiety Symptom Networks over Time. Depress Anxiety. 2024 Jan 1;2024(1):4393070. doi:10.1155/2024/4393070 PubMed PMID: 40226736. - Delli Colli C, Viglione A, Poggini S, Cirulli F, Chiarotti F, Giuliani A, et al. A network-based analysis anticipates time to recovery from major depression revealing a plasticity by context interplay. Translational Psychiatry 2025 15:1. 2025 Jan 28;15(1):32-. doi:10.1038/s41398-025-03246-1 PubMed PMID: 39875363. |
