# Supplementary material for: Network analysis of mental well-being, psychological inflexibility, and psychopathological symptoms in individuals seeking online psychological support
Source: PLoS One. 2026 Sep 23;21(9):e0344411. doi: 10.1371/journal.pone.0344411 (PMC13600521; doi:10.1371/journal.pone.0344411)
Supplement: Peer review history

# Peer review: Decision Letter 0

**DOI:** 10.1371/journal.pone.0344411.r001

**Authors:** Junya Fujino

4 Jun 2026

Dear Dr.  Dominguez-Rodriguez,

Thank you for submitting your manuscript to PLOS ONE. After careful consideration, we feel that it has merit but does not fully meet PLOS ONE’s publication criteria as it currently stands. Therefore, we invite you to submit a revised version of the manuscript that addresses the points raised during the review process.

Please submit your revised manuscript by Jul 19 2026 11:59PM. If you will need more time than this to complete your revisions, please reply to this message or contact the journal office at plosone@plos.org. When you're ready to submit your revision, log on to https://www.editorialmanager.com/pone/ and select the 'Submissions Needing Revision' folder to locate your manuscript file.

- A letter that responds to each point raised by the academic editor and reviewer(s). You should upload this letter as a separate file labeled 'Response to Reviewers'.
- A marked-up copy of your manuscript that highlights changes made to the original version. You should upload this as a separate file labeled 'Revised Manuscript with Track Changes'.
- An unmarked version of your revised paper without tracked changes. You should upload this as a separate file labeled 'Manuscript'.

If you would like to make changes to your financial disclosure, please include your updated statement in your cover letter. Guidelines for resubmitting your figure files are available below the reviewer comments at the end of this letter.

If applicable, we recommend that you deposit your laboratory protocols in protocols.io to enhance the reproducibility of your results. Protocols.io assigns your protocol its own identifier (DOI) so that it can be cited independently in the future. For instructions see: https://journals.plos.org/plosone/s/submission-guidelines#loc-laboratory-protocols. Additionally, PLOS ONE offers an option for publishing peer-reviewed Lab Protocol articles, which describe protocols hosted on protocols.io. Read more information on sharing protocols at https://plos.org/protocols?utm_medium=editorial-email&utm_source=authorletters&utm_campaign=protocols.

As the corresponding author, your ORCID iD is verified in the submission system and will appear in the published article. PLOS supports the use of ORCID, and we encourage all coauthors to register for an ORCID iD and use it as well. Please encourage your coauthors to verify their ORCID iD within the submission system before final acceptance, as unverified ORCID iDs will not appear in the published article. *Only*  the individual author can complete the verification step; PLOS staff *cannot*  verify ORCID iDs on behalf of authors.

We look forward to receiving your revised manuscript.

Kind regards,

Junya Fujino

Academic Editor

PLOS One

Journal Requirements:

When submitting your revision, we need you to address these additional requirements.

1.Please ensure that your manuscript meets PLOS ONE's style requirements, including those for file naming. The PLOS ONE style templates can be found at

https://journals.plos.org/plosone/s/file?id=wjVg/PLOSOne_formatting_sample_main_body.pdf and

https://journals.plos.org/plosone/s/file?id=ba62/PLOSOne_formatting_sample_title_authors_affiliations.pdf

2. We note that there is identifying data in the Supporting Information file “S1-Data.xlsx”. Due to the inclusion of these potentially identifying data, we have removed this file from your file inventory. Prior to sharing human research participant data, authors should consult with an ethics committee to ensure data are shared in accordance with participant consent and all applicable local laws.

Data sharing should never compromise participant privacy. It is therefore not appropriate to publicly share personally identifiable data on human research participants. The following are examples of data that should not be shared:

-Name, initials, physical address

-Ages more specific than whole numbers

-Internet protocol (IP) address

-Specific dates (birth dates, death dates, examination dates, etc.)

-Contact information such as phone number or email address

-Location data

-ID numbers that seem specific (long numbers, include initials, titled “Hospital ID”) rather than random (small numbers in numerical order)

Data that are not directly identifying may also be inappropriate to share, as in combination they can become identifying. For example, data collected from a small group of participants, vulnerable populations, or private groups should not be shared if they involve indirect identifiers (such as sex, ethnicity, location, etc.) that may risk the identification of study participants.

Additional guidance on preparing raw data for publication can be found in our Data Policy (https://journals.plos.org/plosone/s/data-availability#loc-human-research-participant-data-and-other-sensitive-data) and in the following article: http://www.bmj.com/content/340/bmj.c181.long.

Please remove or anonymize all personal information (<specific identifying information in file to be removed>), ensure that the data shared are in accordance with participant consent, and re-upload a fully anonymized data set. Please note that spreadsheet columns with personal information must be removed and not hidden as all hidden columns will appear in the published file.

3. Your ethics statement should only appear in the Methods section of your manuscript. If your ethics statement is written in any section besides the Methods, please move it to the Methods section and delete it from any other section. Please ensure that your ethics statement is included in your manuscript, as the ethics statement entered into the online submission form will not be published alongside your manuscript.

4. Please include captions for your Supporting Information files at the end of your manuscript, and update any in-text citations to match accordingly. Please see our Supporting Information guidelines for more information: http://journals.plos.org/plosone/s/supporting-information.

5. If the reviewer comments include a recommendation to cite specific previously published works, please review and evaluate these publications to determine whether they are relevant and should be cited. There is no requirement to cite these works unless the editor has indicated otherwise.

Additional Editor Comments:

Dear Dr. Alejandro Dominguez-Rodriguez,

Thank you for submitting your manuscript to PLOS One.

Your manuscript has now been evaluated by two reviewers. Both reviewers found the topic interesting and considered the work to have potential value for the field.

However, they also raised several important points that need to be addressed before the manuscript can be considered further.

In particular, the reviewers request revisions concerning the statistical analysis and the way the results are presented.

Please carefully consider each of the reviewers’ comments and provide a detailed point-by-point response explaining how you have addressed them in the revised manuscript.

Where you choose not to make a suggested change, please provide a clear justification.

I would therefore like to invite you to submit a revised version of your manuscript.

Please ensure that the revised manuscript clearly indicates the changes made, and that the response letter addresses all reviewer comments in a systematic manner.

We look forward to receiving your revised manuscript.

Sincerely,

Junya Fujino

[Note: HTML markup is below. Please do not edit.]

Reviewer's Responses to Questions

**Comments to the Author**

1. Is the manuscript technically sound, and do the data support the conclusions?

Reviewer #1: Yes

Reviewer #2: Yes

**********

2. Has the statistical analysis been performed appropriately and rigorously?

Reviewer #1: No

Reviewer #2: Yes

**********

3. Have the authors made all data underlying the findings in their manuscript fully available?

The PLOS Data policy

Reviewer #1: Yes

Reviewer #2: Yes

**********

4. Is the manuscript presented in an intelligible fashion and written in standard English?

Reviewer #1: Yes

Reviewer #2: Yes

**********

Reviewer #1: The manuscript requires improvement.

Line 194: The statistical analysis section should be clearly divided into the following components: network estimation (Graphical LASSO), network visualization, centrality analysis, and network stability and accuracy (bootstrap)

Line 196: The network structure was estimated using a Graphical Gaussian Model with L1 regularization via graphical LASSO.

Line 199: Usually ≥10 participants per node for more stable estimates.

Line 204: The sentence could be improved e.g. The network was estimated using residualized item scores obtained from linear regression models such as age, gender, education, occupation, and marital status.

Table 1: The nodes represent which outcome measure is to be highlighted in the table footnote.

The statistical test applied in Table 2 should be clearly stated in both the statistical analyses section and the corresponding table footnote

Table 2: The label ‘Overall’ is to be replaced with ‘Total’. Percentage values either based on row or column percentages sum to 100%, according to the intended focus of the table.

Other plots could be provided e.g. Edge-weight accuracy plot (bootstrap), bootstrapped difference test plot etc

Not all references conformed to the journal’s format.

Reviewer #2: In this study, the authors analyzed data from the Online Well-being Intervention. A total of 967 Mexican participants were included. A psychological network comprising 74 items was estimated, and centrality indices, including strength, closeness, and betweenness, were calculated to determine the relative importance of individual elements within the network. The network revealed multiple inter-domain associations, particularly negative relationships between mental well-being and symptoms of depression, anxiety, stress, and psychological inflexibility. Items reflecting self-evaluation and emotional well-being consistently emerged as the most central elements across centrality metrics. These findings are potentially interesting and may be valuable to clinicians. Overall, the manuscript is well written and easy to follow. My comments are as follows:

#1. Study sample and rationale

In the Discussion, the authors state: “The purpose of the present study was to identify key nodes related to stress, anxiety, depression, and psychological inflexibility that may play a central role in mental well-being in a sample of individuals seeking online psychological support. As far as we know, this research is the first to use network analysis to explore how these psychological constructs are connected within this group.” However, the characteristics of the study sample and the rationale for focusing on this population are not sufficiently explained in the Introduction. The authors should provide more detailed information about the sample, including the context in which participants sought online psychological support, and explain more clearly why examining these psychological constructs in this population is important.

#2. Number of nodes

In the Statistical Analyses section, the authors state that the analysis used 967 participants for a network of 74 nodes (N/p ≈ 13.1), exceeding recommended thresholds. However, in the legend for Figure 2, the authors state that the network contains 73 nodes, each representing an item, with edges indicating the magnitude and direction of partial correlations between pairs of nodes while controlling for all other analyzed variables. This discrepancy between 73 and 74 nodes may confuse readers. Please revise the relevant sections to ensure consistency and clearly state the correct number of nodes included in the network analysis.

#3. Interpretation of negative Z-scores in Figure 3

In Figure 3, the authors state that standardized Z-scores, with a mean of 0 and a standard deviation of 1, indicate the importance of each item according to the calculated centrality metric, and that the further a point shifts to the right, the greater its importance. However, some items appear to have negative Z-scores and are located toward the left side of the figure. Please clarify how negative Z-scores should be interpreted in this context.

#4. Network stability

In the Network Stability section, the authors state that the correlation stability (CS) coefficients exceeded the recommended threshold of 0.5. Specifically, the coefficients were 0.75, 0.75, 0.67, and 0.44 for edges, strength, closeness, and betweenness, respectively. However, the CS coefficient for betweenness appears to be 0.44, which does not exceed the 0.5 threshold. Please clarify this point and provide a more detailed explanation of this issue.

#5. References and citation support

Some statements in the manuscript appear to require stronger citation support. Please review the manuscript carefully and add appropriate references where necessary. For example, the following statement in the Introduction should be supported by relevant literature: “It has been proposed that the concept of mental health or well-being refers to a stable state within a symptom network characterized by weak interconnections among its elements.”

**********

what does this mean?). If published, this will include your full peer review and any attached files.

If you choose “no”, your identity will remain anonymous but your review may still be made public

**Do you want your identity to be public for this peer review?**  For information about this choice, including consent withdrawal, please see our Privacy Policy

Reviewer #1: No

Reviewer #2: No

**********

[NOTE: If reviewer comments were submitted as an attachment file, they will be attached to this email and accessible via the submission site. Please log into your account, locate the manuscript record, and check for the action link "View Attachments". If this link does not appear, there are no attachment files.]

To ensure your figures meet our technical requirements, please review our figure guidelines: https://journals.plos.org/plosone/s/figures

You may also use PLOS’s free figure tool, NAAS, to help you prepare publication quality figures: https://journals.plos.org/plosone/s/figures#loc-tools-for-figure-preparation.

NAAS will assess whether your figures meet our technical requirements by comparing each figure against our figure specifications.

# Author comment: Author response to Decision Letter 1

**DOI:** 10.1371/journal.pone.0344411.r002

14 Aug 2026

The response to the reviewers is attached in the document: Response to the reviewers.

Attachment Submitted filename: Response to reviewers.docx

# Peer review: Decision Letter 1

**DOI:** 10.1371/journal.pone.0344411.r003

**Authors:** Junya Fujino

30 Aug 2026

Network Analysis of Mental Well-being, Psychological Inflexibility, and Psychopathological Symptoms in Individuals Seeking Online Psychological Support

PONE-D-26-08989R1

Dear Dr. Dominguez-Rodriguez,

We’re pleased to inform you that your manuscript has been judged scientifically suitable for publication and will be formally accepted for publication once it meets all outstanding technical requirements.

Within one week, you’ll receive an e-mail detailing the required amendments. When these have been addressed, you’ll receive a formal acceptance letter and your manuscript will be scheduled for publication.

An invoice will be generated when your article is formally accepted. Please note, if your institution has a publishing partnership with PLOS and your article meets the relevant criteria, all or part of your publication costs will be covered. Please make sure your user information is up-to-date by logging into Editorial Manager at Editorial Manager® and clicking the ‘Update My Information' link at the top of the page. For questions related to billing, please contact billing support.

If your institution or institutions have a press office, please notify them about your upcoming paper to help maximize its impact. If they’ll be preparing press materials, please inform our press team as soon as possible -- no later than 48 hours after receiving the formal acceptance. Your manuscript will remain under strict press embargo until 2 pm Eastern Time on the date of publication. For more information, please contact onepress@plos.org.

Kind regards,

Junya Fujino

Academic Editor

PLOS One

Additional Editor Comments (optional):

Reviewers' comments:

Reviewer's Responses to Questions

**Comments to the Author**

Reviewer #1: (No Response)

Reviewer #2: All comments have been addressed

**********

2. Is the manuscript technically sound, and do the data support the conclusions?

Reviewer #1: (No Response)

Reviewer #2: (No Response)

**********

3. Has the statistical analysis been performed appropriately and rigorously?

Reviewer #1: (No Response)

Reviewer #2: (No Response)

**********

4. Have the authors made all data underlying the findings in their manuscript fully available?

The PLOS Data policy

Reviewer #1: (No Response)

Reviewer #2: (No Response)

**********

5. Is the manuscript presented in an intelligible fashion and written in standard English?

Reviewer #1: (No Response)

Reviewer #2: (No Response)

**********

Reviewer #1: (No Response)

Reviewer #2: (No Response)

**********

what does this mean?). If published, this will include your full peer review and any attached files.

If you choose “no”, your identity will remain anonymous but your review may still be made public.

**Do you want your identity to be public for this peer review?**  For information about this choice, including consent withdrawal, please see our Privacy Policy

Reviewer #1: No

Reviewer #2: No

**********

# Editor report: Acceptance letter

**DOI:** 10.1371/journal.pone.0344411.r004

**Authors:** Junya Fujino

PONE-D-26-08989R1

PLOS One

Dear Dr. Dominguez-Rodriguez,

I'm pleased to inform you that your manuscript has been deemed suitable for publication in PLOS One. Congratulations! Your manuscript is now being handed over to our production team.

At this stage, our production department will prepare your paper for publication. This includes ensuring the following:

* All references, tables, and figures are properly cited

* All relevant supporting information is included in the manuscript submission,

* There are no issues that prevent the paper from being properly typeset

You will receive further instructions from the production team, including instructions on how to review your proof when it is ready. Please keep in mind that we are working through a large volume of accepted articles, so please give us a few days to review your paper and let you know the next and final steps.

Lastly, if your institution or institutions have a press office, please let them know about your upcoming paper now to help maximize its impact. If they'll be preparing press materials, please inform our press team within the next 48 hours. Your manuscript will remain under strict press embargo until 2 pm Eastern Time on the date of publication. For more information, please contact onepress@plos.org.

You will receive an invoice from PLOS for your publication fee after your manuscript has reached the completed accept phase. If you receive an email requesting payment before acceptance or for any other service, this may be a phishing scheme. Learn how to identify phishing emails and protect your accounts at https://explore.plos.org/phishing.

If we can help with anything else, please email us at customercare@plos.org.

Thank you for submitting your work to PLOS One and supporting open access.

Kind regards,

PLOS One Editorial Office Staff

on behalf of

Dr. Junya Fujino

Academic Editor

PLOS One
